# Toileting Behaviors Related to Urination in Women: A Scoping Review

**DOI:** 10.3390/ijerph16204000

**Published:** 2019-10-19

**Authors:** Chen Wu, Kaikai Xue, Mary H. Palmer

**Affiliations:** 1 School of Nursing, University of North Carolina at Chapel Hill, Chapel Hill, NC 27599, USA; wuchen@email.unc.edu; 2 School of Nursing, Xuzhou Medical University, Xuzhou 221000, China; cathyxuekaikai@hotmail.com

**Keywords:** female, urination, lower urinary tract symptoms, urinary bladder, posture

## Abstract

This scoping review explores the state of science regarding women’s toileting behaviors, gaps in knowledge, and areas for future research. Online databases were searched to identify papers published in English between January 2010 through July 2019; the search identified 25 articles. The Toileting Behaviors–Women’s Elimination Behaviors scale has been published in four validated language versions and used in 17 of the 25 studies. The most frequent behaviors include concern about public toilet cleanliness, delaying urination when busy or away from home, and using different toileting postures at and away from home. Determinants of toileting behaviors include environmental factors, chronic health conditions, and cognitive/psychological factors. Associations were found between toileting behaviors and lower urinary tract symptoms and between toileting postures and uroflowmetric parameters and post-void residual volume. Strategies that address modifiable determinants of toileting behaviors should be developed and tested in future research. Furthermore, little is known about the toileting behaviors and bladder health in older women and women from developing countries. Rigorous studies are needed to better understand the underlying mechanisms of toileting behaviors, the nature of associations between toileting behaviors and lower urinary tract symptoms, and effects of the environment on women’s toileting behaviors.

## 1. Introduction

Urination is an essential bodily function and the behaviors that are used to fulfill this function can be instrumental in maintaining women’s health. The bladder stores urine produced by the kidneys and releases it through complex interactions among the central and peripheral nervous systems and lower urinary tract structures (i.e., bladder, bladder neck, sphincters, and urethra). Toileting behaviors that are related to both storing and emptying urine are primarily intra-individual cognitive and emotional processes that are influenced by multiple factors (e.g., interpersonal, social, environmental, and cultural factors), of which the awareness or sensation of a full bladder is only one. For example, women can feel vulnerable to physical and emotional circumstances that are related to removing or adjusting clothing and/or assuming a toileting posture, especially in unfamiliar places outside the home. In such environments, women may decide to delay urination despite the sensation of the need to urinate. Exposure to odors and others’ urine, feces, and/or menstrual blood can create feelings of disgust and increase the sense of vulnerability to being exposed to germs or disease and, as a result, women may alter their usual toileting behaviors.

Until recently, researchers and clinicians have paid little attention to toileting behaviors that usually occur in private locations and that women rarely discuss publicly. The reasons for the lack of research and clinical attention or interest are unclear, especially in light of interest in children’s behaviors when being toilet-trained and the widespread use of behavioral interventions (e.g., bladder training, habit training, and prompted voiding) to treat or manage urinary incontinence (UI) and overactive bladder (OAB).

Recent attempts to better understand toileting behaviors include the development of a conceptual framework of women’s toileting behaviors that include the following five domains: (1) women’s preferences for places to urinate, (2) urinating in the absence of the sensation of the need to urinate, (3) delaying urination in the presence of the sensation of the need to urinate, (4) straining or pushing down to urinate, and (5) preferences for the positions or stances to urinate [1]. Furthermore, research over the past decade has revealed that some toileting behaviors are associated with specific lower urinary tract symptoms (LUTS), especially UI and OAB [2,3]. Thus, documenting the current state of knowledge about toileting behaviors and identifying gaps in knowledge are important to advance research into women’s lower urinary tract health. Three specific questions were used to explore the state of the science regarding women’s toileting behaviors:

(1) How are toileting behaviors measured?

(2) What are the toileting behaviors (and their determinants) of women aged 18 years old and older?

(3) What are the outcomes of the various toileting behaviors?

## 2. Methods

The authors carried out this scoping review in accordance with methodology for scoping reviews published by the Joanna Briggs Institute that includes the PCC search strategy, i.e., Participants, Concepts, and Context, and in accordance with the checklist of PRISMA (Preferred Reporting Items for Systematic Reviews and Meta-Analyses) Extension for Scoping Reviews (PRISMA-ScR) [4,5].

### 2.1. Inclusion and Exclusion Criteria

Inclusion criteria for the articles reviewed were (1) adult women 18 years old and older were study participants; (2) information about the timing of urination (i.e., delaying or prematurely urinating) and the place and posture/position for urination and/or nature of urination (i.e., straining to urinate) is included; (3) the study methods used are qualitative and/or quantitative; and (4) the studies were published in English-language peer-reviewed journals. Exclusion criteria for the articles reviewed were (1) articles written as reviews, concept analyses, commentaries, case studies, dissertations, or theses and (2) conference papers or posters that report the same findings as published in peer-reviewed papers.

### 2.2. Search Strategy

In consultation with a Health Sciences Library librarian, the authors searched three databases: PubMed, the Cumulative Index to Nursing and Allied Health Literature (CINAHL), and Web of Science. The librarian helped the authors conduct a pilot search in PubMed to analyze keywords and index terms that describe relevant studies and to apply these keywords and index terms to search for studies across the three target databases. All searches were set for January 2010 through July 2019. A concept analysis of toileting behaviors [1] summarized the literature prior to 2010, thus this timeframe is consistent with the publication of emerging studies that focused exclusively on toileting behaviors in women. The English language was used as a filter for all three databases. The Table S1 provides details about the search strategies.

### 2.3. Screening Strategy

Two of the authors (CW and KKX) conducted the initial data screening via Covidence (www.covidence.org). They reviewed the titles and abstracts of all the studies and, if the information in the abstracts was unclear for determining eligibility, reviewed the full article text. During the data screening phase, several face-to-face meetings of all three authors were held to discuss and resolve any disagreements and, when applicable, at least one author made direct contact with the author(s) of the reviewed studies to obtain clarification and resolve outstanding issues or answer the researchers’ questions. The authors of two published conference abstracts were contacted; one of those authors did not respond, so that abstract was removed from this review.

### 2.4. Information Extracting and Synthesis Strategy

The authors developed a draft charting form based on the Joanna Briggs Institute methodology. After pilot testing this tool using a sample of three studies (i.e., one methodological study, one determinants of toileting behavior study, and one outcomes related to toileting behavior study), the authors modified the charting form and then used it to extract data from all the eligible studies. Two authors (CW and KKX) performed the initial data extraction for the following categories: first author, year of publication, country of origin, journal, design, study population, sample size, aims, and key findings. Two authors (CW and MHP) independently double-checked all the data extraction entries. The authors then created tables using the information that corresponded to the research questions under review. The findings are presented using a narrative approach that includes numerical analyses (i.e., analyzing the nature and extent of the studies by using tables and charts) and thematic construction (i.e., thematic findings organized according to the analytic questions proposed) [6].

## 3. Results

### 3.1. Sample Characteristics

**Figure 1:**
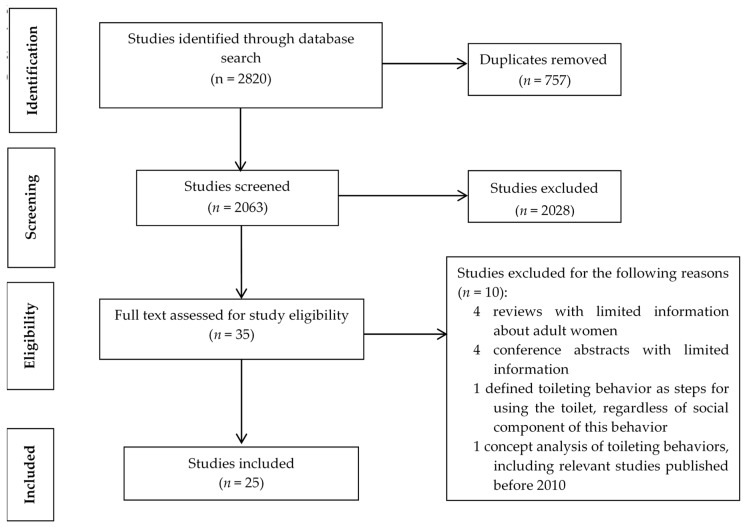
PRISMA flow diagram for inclusion of articles in scoping review.

This review includes a total of 25 articles; see Figure 1 for a flow diagram of the process for inclusion of articles in this scoping review. The authors located one randomized controlled trial (RCT), one repeated measures study, three methodological studies, three qualitative studies (i.e., semi structured interviews and focus groups), and 17 cross-sectional studies. Seven studies reported using online questionnaires [7,8,9,10,11,12,13], 14 used a field survey or postal delivered questionnaire [2,3,14,15,16,17,18,19,20,21,22,23,24,25], and one study combined both a field survey and online questionnaire to collect information [26]. Three studies used uroflowmetry and sonography to measure multiple uroflowmetric parameters (e.g., maximum flow rate, Qmax and average flow rate, and Qave) and used postvoid residual (PVR) urine volume as one of the outcomes [14,15,21]; see Table S2 for an overview of the studies reviewed.

All the reviewed studies included adult women as participants and three studies [3,22,23] included men. Sample sizes for the three qualitative studies ranged from 25 to 96 women [27,28,29], and 104 women participated in the RCT [23]. Sample sizes for the 17 cross-sectional studies ranged from 17 to 6695 women, with small samples (i.e., 17 to 45 women) for three of these studies that included uroflowmetry and sonography measures [14,15,21]. Sample sizes for the three methodological studies ranged from 250 to 321 women [7,16,25].

Clinical nurses and/or midwives and advanced practice providers were enrolled in seven studies [3,8,17,18,24,26,27], nonpregnant women working in academic medical centers were enrolled in three studies [7,9,10], and full-time working women without a specific job description were enrolled in one study [11]. The mean age of these women ranged from 30.2 to 51.2 years old and standard deviations ranged from 6.8 to 13.6 years old.

College/university students were enrolled in three studies [2,12,14], and the mean age of these students ranged from 20.5 to 23.2 years old with standard deviations within ± 4.6 years. Women attending endocrinology clinics were enrolled in two studies. The mean age was not differentiated by sex, and the mean ages for women and men were 59.1 ± 11.7 years old and 66.4 ± 8.2 years old, respectively [22,23]. Other single studies included pregnant women (mean age not reported) [21], elderly women with knee osteoarthritis (mean age 65 ± 4.6 years old) [15], and women attending a Urogynecology Clinic (mean age 60.4 years old) [19]. Women with and without LUTS were enrolled in another six studies: three qualitative studies with women’s mean ages of 42.3 ± 12.6 years old [27], 68 ± 13.4 years old [28], and 60 years old (ranging from 21 to 90 years old) [29] and three quantitative studies with women’s mean ages not reported in one study [16] and the mean ages in the other two studies of 21.6 years old [20] and 41.4 ± 15 years old [13].

### 3.2. Measures of Toileting Behaviors

#### 3.2.1. Available Measures

**Figure 2:**
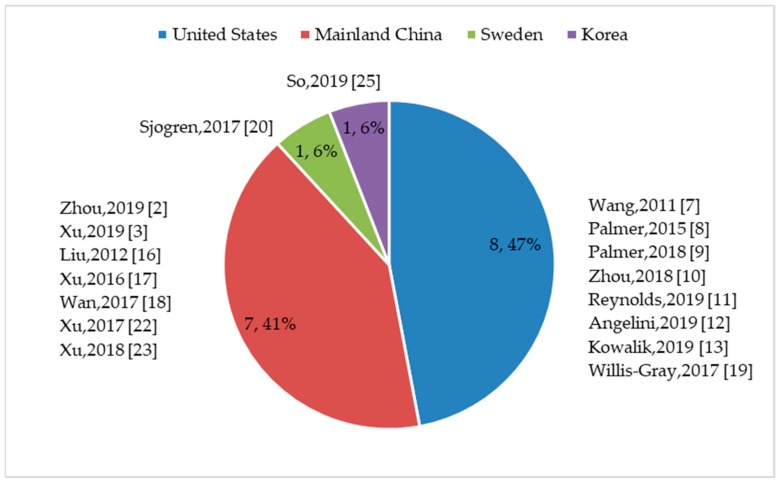
Numbers and percentages of studies where Toileting Behavior–Women’s Elimination Behaviors (TB–WEB scale was used, by country (n, %).

Authors of 19 quantitative studies reported that they used established measures or individual questionnaire items to quantify women’s toileting behaviors [2,3,8,9,10,11,12,13,14,15,17,18,19,20,21,22,23,24,26]. One study used two questionnaire items; the first item included multiple choice questions to elicit information about toileting behaviors used at work and the other item queried the adequacy of bathrooms at work [24]. One study employed three questionnaire items; the first item included multiple choice questions to track how often the participants delayed passing urine at work and the second and third items queried participants’ access to a toilet whenever needed and frequency of reducing fluid intake to delay or avoid passing urine at work [26]. Most studies (*n* = 17) used the Toileting Behavior–Women’s Elimination Behaviors (TB–WEB) scale [2,3,7,8,9,10,11,12,13,16,17,18,19,20,22,23,25]. The TB–WEB scale was used across countries and groups of women (see Figure 2), with most studies conducted in the United States (8/17) [7,8,9,10,11,12,13,19] and China (7/17) [2,3,16,17,18,22,23]. In addition to the three methodological papers that employed the TB–WEB scale [7,16,25], 12 cross-sectional studies that aimed to describe the prevalence and/or explore associations with other variables [2,3,8,9,10,11,13,17,18,19,22,23] and two cross-sectional studies that aimed to describe the prevalence of toileting behaviors and/or explore associations with other variables [12,20] also used the TB–WEB scale; see Table S2 for an overview of the studies reviewed.

#### 3.2.2. TB–WEB Scale Development and Application

Four language versions of the TB–WEB scale were used for the reviewed studies: English, Chinese, Swedish, and Korean (see Table 1). Since its development in 2011 [7], the English language version of the TB–WEB scale has evolved from 18 items to 22 items, with four pictorial and written descriptions of voiding postures for each of two environments: at home and away from home [8,9,10,11,12,13,19]. The Chinese language version of the TB–WEB scale has evolved from 14 items to 22 items for female respondents [2,16,17,18] and now has 15 items for both male and female respondents [3,22,23]. All four of the language versions maintain the original five-domain structure of the TB–WEB scale (i.e., place preference, premature voiding, delayed voiding, postures used to void, and straining to void), with a few exceptions. Four studies that used the Chinese language version of the TB–WEB scale did not include position preference [3,16,22,23], three studies included both male and female respondents [3,22,23], and one study included community-dwelling women [16]. Furthermore, a study conducted at Chinese colleges/universities used a modified version of the TB–WEB scale that included six domains; the additional domain is ‘emptying bladder completely’ [2].

Although the original five-point scale format of ‘never’, ‘rarely’, ‘sometimes’, ‘often’, and ‘always’ remained unchanged, 13 cross-sectional studies collapsed responses into two-point or three-point formats. For example, responses of ‘never’ and ‘rarely’ were collapsed into one category (*n* = 5) [3,17,19,22,23], ‘sometimes’, ‘often’, and ‘always’ were collapsed into one category (*n* = 3) [13,19,20], ‘often’ and ‘always’ were collapsed into one category (*n* = 9) [2,3,8,9,10,11,17,22,23], and ‘rarely’, ‘sometimes’, ‘often’, and ‘always’ were collapsed into one category (*n* = 1) [12]. One cross-sectional study retained the original five-point scale format [18].

The English language version of the TB–WEB scale had acceptable construct and criterion validity and demonstrated similar acceptable reliability (i.e., Cronbach’s α coefficient > 0.7) for all domains [13,18], except in one study where α = 0.582 was reported for position preference for urination [18]. The 14-item Chinese language version and 17-item Korean language version of the TB–WEB scale had acceptable reliability for domains and construct validity, and the Korean language version also had acceptable criterion-related validity [26,29]. The 19-item Swedish language version had acceptable construct validity and acceptable reliability for all domains (α > 0.7), except for position preference for voiding (α = 0.540) [20]. Of the studies in which the TB–WEB scale was administered [2,3,8,9,10,11,13,17,18,19,22,23], only two studies reported reliability [2,22], and one of those reported reliability for the entire scale rather than reporting reliability for each domain [22], see Table 1.

### 3.3. Toileting Behaviors and Their Determinants

#### 3.3.1. Place Preference and Position Preference for Voiding

With regard to place preference, three studies reported that 83% students from one private college in the northeastern United States, 36.4% students from colleges/universities in China, and 33% operating room nurses avoided using public toilets [2,3,12]. Of students at a university in Sweden, 45.5% always emptied their bladder at home to avoid using a public toilet [20]. In one study, proportionally more incontinent women did not avoid public toilets than continent women (27.3% vs. 9.6%, *p =* 0.041) [8]. Furthermore, worry about public toilet cleanliness under the domain of place preference was common. For example, many Chinese clinical nurses (52%~69.5%), students at one private American college (92.2%), students at colleges/universities in China (52.7%), and female employees at an academic medical center (39.4%) often or always worried about the cleanliness of public toilets [2,3,9,12,17]. Most (87.2%) students at a university in Sweden at least sometimes worried about the cleanliness of public toilets [20].

With regard to position preference, in one study, 32.1% of clinical nurses reported crouching/hovering over the toilet when they used toilets away from home [17]. In another study, 24.4% of students reported hovering over the toilet at least sometimes [20]. Willis-Gray and colleagues reported that significantly more women who were attending urogynecology clinics and who had UI crouched or hovered over the toilet to urinate when away from home compared to those who were continent (17.6% vs. 3.8%, *p* = 0.02) [19].

#### 3.3.2. Premature and Delayed Voiding

Many women reported premature voiding, i.e., urinating without a clear sensation of the need to urinate, as indicated in eight studies [3,8,9,12,17,19,20,22]. Furthermore, 80% of employees at an academic medical center [9], 22% of nurses [3], 72.2% of students at colleges/universities in China [2], 97% of students at one private American college emptied their bladders before leaving home [12], 46.5% of students at a university in Sweden emptied their bladder without desire at home and ‘just in case’ [20], and 39% [3] and 44.2% [17] of nurses emptied their bladders without the sensation of the need to urinate before going to sleeping. Proportionally more women with UI emptied their bladder without the sensation of the need to urinate both at home and away from home than continent women: 56.5% vs. 75%, *p* = 0.043 for responses to nonpremature voiding away from home; 47.9% vs. 32.1%, *p* = 0.05 for responses to premature voiding at home; 28.8% vs. 11.1%, *p* = 0.01 for response to premature voiding away from home [8,19].

A wide percentage range of women (25.3%–85.1%) reported delaying urination when they were busy [2,3,9,13,17,20]. When in the workplace, 77.1% of nurses and midwives delayed voiding and 26.9% limited their fluid intake to delay voiding [26]. Proportionally more urinary incontinent women reported waiting too long to urinate compared to continent women in two studies: 52.7% vs. 26.9%, *p* = 0.006 and 41.5% vs. 21.6%, *p* = 0.02 [8,19]. Most students (81.9%) at a private American college reported that they would hold urine when they were away from home [12].

#### 3.3.3. Straining to Void

When at work, proportionally more women with reported UI compared to continent women reported straining to start urination (32.6% vs. 16.7%, *p* = 0.03), keep urine flowing (43.9% vs. 20.4%, *p* = 0.01), empty their bladder completely (49.3% vs. 25.9%, *p* = 0.01), and empty their bladder quickly (37.4% vs. 17.0%, *p* = 0.01) [19]. Many students (72.3%) at a private American college reported straining to empty their bladder quickly [12], 38.8% and 42.2% of students at a university in Sweden strained to empty their bladder completely and strained to void quickly, respectively [27], and less than 20% of students at colleges/universities in China reported straining to urinate [2].

#### 3.3.4. Determinants of Toileting Behaviors

The determinants of toileting behaviors that were identified in the quantitative studies include the TB–WEB scale items that address behaviors that women use when at home and away from home. Women’s life stage [12,15], incontinence status [8,9,16,19], history of a chronic condition, i.e., osteoarthritis and diabetes mellitus [15,22,23], occupational factors (i.e., stress and employer restrictions) [3,11], adequacy, accessibility, or satisfaction with regard to workplace restrooms [16,24,26,27], and health beliefs [17] served as potential determinants of toileting behaviors; see Table S2.

Several themes that may be potential determinants were identified in three qualitative studies that focused on (1) the workplace [27], (2) the nature of toileting and toilets [28], and (3) temporal and cognitive mechanisms of toileting behavior [29]. One of these studies conducted focus groups among nurses and midwives and identified work-related issues that affect urinary symptoms that are related primarily to delayed urination. These issues include a ‘patient first’ culture, nursing team relationships, job demands, and inadequate amenities in the workplace [27]. In the second of the three studies, focus groups of women with and without urinary symptoms that explored reasons for toileting behaviors identified four themes: (1) cues/triggers/alerts that women use to find and use toilets, (2) toilet cleanliness away from and at home, (3) toileting as a nuisance, and (4) situational awareness [28]. The third study explored the relationship between women’s conscious decision-making and bladder sensation that influenced the timing and location for urination. Themes developed from transcripts of semi-structured interviews with women who reported no urinary problems included temporal and cognitive maps, risk issues, habituation, opportunistic behavior, and full bladder awareness [29].

### 3.4. Outcomes of Toileting Behaviors

Twelve studies investigated the association between toileting behaviors and types of LUTS [2,3,7,8,9,11,12,18,20,22,25,26]; see Table S2. Delayed voiding was associated with urinary frequency [20], urinary urgency [9], UI [2,7,8,20], and OAB [3]. Premature voiding was associated with urinary frequency UI [20] and OAB [22]. Straining to void was associated with urinary frequency [20], UI [2,7,20], and OAB [3,22]. Place preference for voiding was associated with urinary frequency [20] and UI [7]. Position preference for voiding was associated with UI [20]. Two studies confirmed that TB–WEB scale scores were associated with UI evaluation scale scores [12,25]. The TB–WEB scale scores for premature voiding, delayed voiding, and straining to void were each associated with the overall LUTS score in one study [18]. Another study reported significant associations between delayed voiding and urinary symptoms including urinary frequency, discomfort due to the need to delay emptying urine, and urinary urgency [26]. One study that reported on limited bathroom use by women in the workplace also found associations between toileting behaviors and specific LUTS [11].

Three studies reported uroflowmetry results for different toileting postures [14,15,21]. One of these studies was conducted to investigate the effects of three postures (i.e., sitting, semi squatting, and crouching) on urinary flow and PVR volume [14]. For that study, 45 college-aged women were recruited, and a longer delay to urinate (i.e., the elapsed time from ‘begin to urinate’ to actual urination) was noted for women who used the semi squatting position compared to women who used either the sitting or crouching toileting postures [14]. In the second study, older women who had knee osteoarthritis were recruited to evaluate the effect of the standing posture on uroflowmetric outcomes and PVR volume [15]. No significant differences in uroflowmetric outcomes and PVR volume were observed for the standing posture compared to the sitting posture [15]. In the third study, Yang and colleagues evaluated the standing posture (compared to the sitting posture) used by pregnant women in their third trimester and found that PVR volume was significantly less (15.8 mL vs. 27.5 mL, *p* = 0.003) for the standing posture compared to the sitting posture [21].

One RCT with adults who had diabetes found that an educational intervention for toileting behaviors was effective in relieving bladder symptoms, increasing quality of life, and decreasing the probability of developing urgency UI [23].

In summary, the majority of participants in the studies were community-dwelling young to mid-aged women. No studies that included women with disabilities or being of advanced age were located. Most studies used the TB–WEB a questionnaire that was administrated by pen and paper and electronically. Multilevel determinants of toileting behaviors were identified including health status, cognitive appraisal and decision-making about the toilet environment, and external factors that restricted toilet access. Little information was located about knowledge, attitudes and behaviors about toileting and the influence of others, i.e., peers and parents, on the formation of behaviors. The study of toileting outcomes to date has mainly focused on lower urinary tract symptoms.

## 4. Discussion

Until recently, toileting behaviors that women use to meet urinary elimination needs when they are at home and away from home had been an under-studied area in women’s urologic health. This scoping review provides synthesized information about the state of the science, gaps in knowledge about toileting behaviors, the determinants and outcomes of various toileting behaviors, and areas for future research into women’s toileting behaviors.

### 4.1. The TB–WEB Scale

Toileting behaviors are associated with two major functions of the lower urinary tract: storing and emptying urine. In an early concept analysis of toileting behaviors related to urination, the identified attributes were voiding place preference, voiding time related behaviors, voiding position related behaviors, and voiding style related behaviors [1]. Wang and Palmer used this information to develop an 18-item English-language version of the TB–WEB scale. The 18 items were distributed over five domains: premature voiding, straining to void, place preference for voiding, delayed voiding, and position preference for voiding. Each item was rated using a five-point scale: ‘never’, ‘rarely’, ‘sometimes’, ‘often’, and ‘always’ [7]. Subsequently, the TB–WEB scale was adapted for women living in both developed and developing countries and, although the number of items varies in later versions, the five-domain structure generally has been maintained. Acceptable reliability coefficients for each domain, construct validity, and criterion-related validity have been confirmed for all the domains except for position preference for voiding among young women (i.e., about 20 years old). The reliability coefficients reported in the reviewed articles for position preference range from 0.54 to 0.83.

Because sitting-type toilets are predominant in developed countries, the sitting posture is considered the ‘default’ posture. In developing countries such as China, sitting-type toilets are commonly installed in urban homes. However, both non sitting and sitting types of toilets can be found in environments outside homes [30,31]. Moreover, the terms ‘public places’ and ‘places away from home’ may have different connotations, especially for young women. For example, some women may view a coffee shop where they often go to work or study as a familiar public place, whereas other women may perceive it as an unfamiliar place. These differing perceptions could have an impact on how women read and interpret questionnaire items about toileting postures, and these interpretations could introduce judgement bias that guide women’s responses on questionnaire items about toileting postures or positions. This potential bias also may be the reason that some researchers excluded the position preference domain from their study [16]. In addition, judgment bias may explain the low reliability coefficients in studies conducted among college/university students in developed countries [12,20].

Future research should investigate the role of biased responses for position preference items, assumptions and perceptions that women hold about toilet types and their availability, and the terms ‘public places’ or ‘places away from home’. Furthermore, although the TB–WEB scale was developed originally for use exclusively with women, it has been administered to both men and women [3,23]. Revision of items under the position or posture preference domain should be sex-specific because of differences in male and female genitourinary anatomy and types and availability of toilet facilities in public places.

Findings from several studies reveal that the location of toilets, i.e., at home and away from home, affect toileting behaviors. For example, some women who participated in focus groups expressed concern about ‘unfamiliar’ toilets, such as those at rest stops on highways and on airplanes [28]. Further conceptualization of place preference for voiding is needed because toilets ‘away from home’ can be both familiar and unfamiliar to different women. That is, toilets in the workplace or at school where they are used frequently may be perceived and used differently than toilets in places that are not used by women or infrequently used by women. Cleanliness of toilets also is an important issue for many women across age groups and cultures. Evidence shows that women are comfortable with using toilets in friends’ homes [28]. Identifying internal ‘rules’ about toilet cleanliness that influence behaviors will foster better understanding about toileting behaviors and improve refinement of measures for toileting behaviors.

Further conceptualization and measurement of toileting behaviors require the inclusion of specific environmental factors, including resource allocations, e.g., the number of available toilets and provision of toilet paper or not, toilet environment maintenance or not, provision of running water to wash hands or not, and privacy, e.g., locks on stall doors, or even doors at all [32,33]. These factors are likely to affect women’s decision-making about using public toilets by raising health and security concerns and may discourage women from using toilets if they are unsatisfactory.

In addition, the literature includes multiple terms and definitions for positions or postures that women use to urinate, such as semi squatting, sitting, squatting, crouching, and hovering [7,14]. For example, Yang et al. [14] described ‘crouching’ using an image of a woman with her feet on the toilet seat and squatting over the toilet bowl whereas Wang and Palmer [7] described ‘crouching’ using an image of a woman with her feet on the floor and her buttocks not making contact with the toilet seat. To avoid confusion for respondents and to promote comparisons of findings across studies, standardized definitions that include pictorial representations of each position should be employed.

### 4.2. Toileting Behaviors and Their Determinants

Women’s multiple and varied hygienic needs (e.g., urinary, bowel, and menstrual) are sometimes overlooked when designing and planning sanitation systems for public toilets [34]. Based on the reviewed studies, many women report avoiding public toilets, having concerns about their cleanliness, and avoid making contact with public toilet seats by crouching or hovering. Exposing one’s external genitalia to unflushed toilet bowls and/or obvious urine, feces, or menstrual blood on toilet seats left by another person can invoke strong feelings of revulsion [35]. The qualitative evidence provided in this scoping review demonstrates that, even when a public toilet and toilet seat look clean, women remain concerned about uncleanliness and germs [28,29]. This response to ‘dirty’ toilets can be viewed as a motivator to avoid using public toilets and adopt hygienic behaviors to prevent infection and disease [35]. Thus, some women may view non sitting toileting positions as beneficial to their health when they use public sitting toilets. In addition to toilet cleanliness, participants’ own characteristics, including their continence status, are possible determinants of place and position preference for voiding. For example, in some of the reviewed studies, women with incontinence were more likely than continent women not to avoid using public toilets but to use hovering or couching positions when using them. Although place preference and posture preference for voiding appear to be unique concepts, they share similar determinants and are likely correlated.

The timing of urination is another important factor that was investigated in this scoping review. Normal functional bladder capacity in adult females is approximately 300 mL to 400 mL, which evokes a clear sensation of the need to urinate [29,36]. A wide percentage range of women who were participants in the reviewed studies reported that they often/always urinated without the sensation of the need to void. For example, 22% to 97% of working women and college students reported voiding before leaving their home without having the need to urinate, and 39% to 44.2% of clinical nurses reported this same toileting behavior before going to sleep. In addition, 25.3% to 85.1% of women reported delayed voiding, indicating the intentional suppression of, or ignoring the sensation of, the need to void. The prolonged time interval between the conscious awareness of bladder filling and the clear and compelling sensation of the need to urinate may be due to contextual and cognitive considerations [29]. As one example, a college-aged woman may drink a 360 mL serving of a carbonated beverage at lunch before an hour-long test, but because of a long queue at the restroom and the absence of the clear sensation of the need to void, she consciously delays voiding before the test only to have a proximate compelling need to void that distracts her attention away from concentrating on the exam.

Opportunism or cues (e.g., walking past a water fountain or restroom), anticipation of the availability and quality of public toilets, experience/calculation of the next need to use the toilet, and occupational factors (e.g., stress or restrictions) could lead women not to wait for, or to ignore, the clear sensation of the need to urinate, resulting in premature voiding or delayed voiding. Therefore, premature voiding and delayed voiding likely are correlated concepts. Although these behaviors may derive from the same pathway, they are demonstrably different. In addition, some evidence suggests that women with UI are likely to ignore the clear sensation of the need to urinate. More research is warranted to understand the underlying mechanisms of toileting behaviors.

Limited evidence is available regarding the determinants for straining to void. Only one study indicated that being in the workplace and having UI may serve as determinants of straining to void [19]. Straining to void was more prevalent in college/university students in developed countries than students in a developing country, but the reasons for this finding are not clear. Cultural differences in the use and understanding of the terms “strain” or “straining” may exist and should be studied. In addition, research that investigates the etiology and maintenance of this behavior among groups of women across cultures is needed.

### 4.3. Outcomes of Toileting Behaviors

Toileting behaviors are not consistently associated with all types of LUTS. Delayed voiding, premature voiding, and straining to void are associated with UI, OAB, and urinary frequency. Delayed voiding is also associated with the symptom of urinary urgency. Place preference for voiding is associated with UI and urinary frequency, and position preference for voiding is associated with UI and some quantitative outcomes, i.e., uroflowmetric parameters and PVR volume. Some evidence from the reviewed studies confirms significant associations between the overall score of toileting behaviors and UI [12,15], between toileting behaviors scores and overall LUTS score [18], and between delayed voiding and urinary symptoms (e.g., urinary frequency and urinary urgency) [26].

Three issues that should be addressed in future studies will further increase researchers’ confidence in developing potential toileting behavioral interventions. First, the relationship between toileting behaviors and specific LUTS is still unclear. Most of the findings from this scoping review were derived from cross-sectional studies. Prospective cohort studies are needed to track toileting behaviors and LUTS to determine their relationships. Second, linear/logistic regression, which assumes little or no multicollinearity among independent variables, was used in the reviewed studies to test associations. Although the evaluation of statistical analysis rigor falls outside the scope of scoping reviews, some potential violations of statistical assumptions may be present in the reviewed studies. Structural equation modeling, which allows for dependency among independent variables, may offer a better option for future studies. Last, most evidence indicates that some toileting behaviors are not consistently associated with specific LUTS. An overall sum score from a multidimensional scale cannot adequately reflect the separate domains, and associations between the overall sum score of independent variables and the overall sum score of dependent variables should be avoided in order to advance research beyond merely descriptive or correlational studies.

Studies of toileting behaviors were unevenly distributed across the female life course with most studies focused on working women and college/university students. Although the age range is wide, quantitative studies focused mainly on adult women aged younger than 60 years, whereas qualitative studies focused mainly on older women. Studies that employ a life course approach should help to alleviate gaps in knowledge.

## 5. Conclusions

Toileting behaviors are important factors to measure when investigating women’s bladder health. To date, the TB–WEB scale has been the most widely used tool to quantify women’s toileting behaviors. Explicit and consistent descriptions of voiding postures and place preferences are still needed. This review reveals that the sensation of ‘needing to void’ is not the only factor that drives the behaviors associated with urination. Many cognitive factors, e.g., situational awareness and (dis)satisfaction about toilets, and environmental factors, e.g., employer restrictions and (in)adequacy of toilets, along with women’s own health conditions are likely to mediate toileting behaviors. Clearly, toileting behaviors cannot be viewed in isolation from physical and social environments. More studies are needed in developed and developing countries across different age cohorts to determine these macro-level effects on women’s toileting behaviors. Furthermore, the nature of the relationship between toileting behaviors and bladder health requires further exploration to further the science of women’s health.

## Figures and Tables

**Table 1:** Toileting Behaviors of Women, Measured Using TB–WEB Scale (*n* = 17).

First Author (Year) Country	Sample (Female unless Otherwise Noted, Age Defined as Average Age ± Standard Deviation, unless Otherwise Noted)	Version of TB–WEB Scale	Domain (Number of Items): Scores of Domains within Score Range of 1–5	Scaling Format of Item Frequency	Psychometric Characteristics	
					Reliability	Validity
Zhou (2019) [2] China	College/university students Age: 20.5 ± 1.6 years	22-item Chinese version	Premature voiding (5) Straining to void (4) Place preference for voiding (4) Delayed voiding (3) Position preference for voiding (4) Emptying bladder completely (2)	‘Often’ and ‘always’ were summarized as habitual behavior.	Cronbach’s alpha for each dimension was more than 0.7. Overall Cronbach’s alpha was 0.80.	N/A
Xu (2019) [3] China	Operating room nurses. Males were included in the sample. Overall age: 30.2 ± 7.1 years; female age unknown	15-item Chinese (male/female) version	Both male and female participants: Premature voiding (4): 2.3 ± 0.9 Straining to void (4): 2.2 ± 0.9 Place preference for voiding (4): 3.0 ± 1.0 Delayed voiding (3): 3.3 ± 0.9	Never/rarely Sometimes Often/always	Not reported	N/A
Wang (2011) [7] USA	Employees of a large university and academic medical center Age: 51.2 ± 6.8 years	18-item English version	Premature voiding (5): 1.75 ± 0.11 Straining to void (4): 1.91 ± 0.06 Place preference for voiding (4): 2.81 ± 0.61 Delayed voiding (3): 2.79 ± 0.25 Position preference for voiding (2): 2.01 ± 0.16	N/A	Cronbach’s alpha: Premature voiding (α = 0.88) Straining to void (α = 0.86) Place preference for voiding (α = 0.71) Delayed voiding (α = 0.70) Position preference for voiding (α = 0.73)	Construct validity: Five subscales explained 67% variance of latent toileting behaviors. Criterion-related validity: Incontinent vs. continent group: straining (t = 2.12, *p* < 0.05), place preference (t = 2.24, *p* < 0.05), delayed (t = 2.70, *p* < 0.01), position preference (t = 2.07, *p* < 0.05). Total scale (t = 3.79, *p* < 0.01) is significant and premature voiding is nonsignificant (t = 1.39, *p* > 0.05).
Palmer (2015) [8] USA	Advanced practice providers Age: 45.1 ± 10.6 years	19-item English version	Premature voiding (5) Straining to void (4) Place preference for voiding (4) Delayed voiding (3) Position preference for voiding (3)	Never Rarely/sometimes Often/always	Not reported	N/A
Palmer (2018) [9] USA	Employees of a large academic medical center Age: 47.3 ± 13.6 years	22-item English version	Premature voiding (5) Straining to void (4) Delayed voiding (3) Place preference for voiding (4) Position preference (4) Two additional items tested the intentional or unintentional complete emptying of bladder.	‘Often/always’ was regarded as having the behavior, otherwise, not having the behavior.	Not reported	N/A
Zhou (2018) [10] USA	Employees of a large academic medical center Age: 46.9 ± 12.9 years	22-item English version	Premature voiding (5) Straining to void (4) Delayed voiding (3) Place preference for voiding (4) Position preference (4) Two additional items tested the intentional or unintentional complete emptying of bladder.	‘Often/always’ was regarded as having the behavior, otherwise, not having the behavior.	Not reported	N/A
Reynolds (2019) [11] USA	Full-time working women Age: 38.7 ± 12.2 years	19-item English version	Premature voiding (5) Straining to void (4) Place preference for voiding (4) Delayed voiding (3) Position preference for voiding (3)	‘Often’ and ‘always’ were summarized as habitual behavior.	Not reported	N/A
Angelini (2019) [12] USA	Private college undergraduates Age: 21.2 ± 0.46 years	18-item English version	Premature voiding (5) Straining to void (4) Place preference for voiding (4) Delayed voiding (3) Position preference for voiding (2)	‘Rarely’, ‘sometimes’, ‘often’, and ‘always’ were summarized as having the behavior.	Cronbach’s alpha: 0.846 for entire scale Premature voiding (α = 0.868) Straining to void (α = 0.919) Place preference for voiding (α = 0.731) Delayed voiding (α = 0.834) Position preference for voiding (α = 0.582)	Construct validity: Four subscales explained 72.8% variance of latent toileting behavior. Convergent and discriminant validity: Confirmed by establishing significant associations between toileting behaviors and stress urinary incontinence (*r* = 0.293, *p* = 0.015), urgency urinary incontinence (*r* = 0.342, *p* < 0.001), stress urinary incontinence related to sport or physical activity (*r* = 0.350, *p* < 0.001), and urinary urgency (*r* = 0.334, *p* < 0.001).
Kowalik (2019) [13] USA	Living in a community Age: 41.4 ± 15 years	24-item English version	Convenience voiding (5) Straining to void (4) Place preference for voiding (4) Delayed voiding (3) Position preference for voiding (8)	‘Sometimes’, ‘often’, and ‘always’ were regarded as having the behavior, otherwise, not having the behavior.	Not reported	N/A
Liu (2012) [16] China	Living in a community and reported having urinary incontinence Age: unknown	14-item Chinese version	Premature voiding (4) Straining to void (4) Place preference for voiding (3) Delayed voiding (3)	N/A	Cronbach’s alpha: Premature voiding (α = 0.92) Straining to void (α = 0.95) Place preference for voiding (α = 0.75) Delayed voiding (α = 0.8)	Construct validity: Four subscales explained 79% variance of latent toileting behaviors.
Xu (2016) [17] China	Clinical nurses from three hospitals Age: 30.6 ± 7.9 years	17-item Chinese version	Premature voiding (4): 2.30 ± 0.92 Straining to void (4): 1.89 ± 0.87 Place preference for voiding (4): 2.90 ± 0.84 Delayed voiding (3): 3.12 ± 0.77 Position preference for voiding (2): 2.45 ± 1.06	Never/rarely Sometimes Often/always	Not reported	N/A
Wan (2017) [18] China	Clinical nurses Age: 30.6 ± 7.9 years	17-item Chinese version	Premature voiding (4): 2.30 ± 0.92 Straining to void (4): 1.89 ± 0.87 Place preference for voiding (4): 2.90 ± 0.84 Delayed voiding (3): 3.12 ± 0.77 Position preference for voiding (2): 2.45 ± 1.06	Never Rarely Sometimes Often Always	Not reported	N/A
Willis-Gray (2017) [19] USA	Patients in a urogynecology clinic Age: mean = 60.4 years	22-item English version, with 4 pictorial images	Premature voiding (5) Straining to void (5) Attitudes towards public bathrooms (4) Delayed voiding (4) Position preference for voiding (4) Four pictorial descriptions: sitting, crouching, squatting, or standing	Never, rarely, or never/rarely Unreported Sometimes/often/always	Not reported	N/A
Sjogren (2017) [20] Sweden	University students Age: mean = 21.6 years, ranging from 18 to 25	19-item Swedish version	Premature voiding (5): 2.0 ± 0.8 Straining to void (4): 1.9 ± 0.8 Place preference for voiding (4): 3.63 ± 0.85 Delayed voiding (3): 2.63 ± 0.83 Position preference for voiding (3): 1.1 ± 0.47	Never Rarely Sometimes Often Always The latter three options were summarized as ‘at least sometimes’.	Cronbach’s alpha: Premature voiding (α = 0.84) Straining to void (α = 0.82) Place preference for voiding (α = 0.81) Delayed voiding (α = 0.71) Position preference for voiding (α = 0.54)	Construct validity: Five subscales explained 66% variance of latent toileting behaviors.
Xu (2017) [22] China	Participants from an endocrinology department in one hospital who had Type 2 diabetes mellitus. Men were included in the sample. Overall age: 59.1 ± 11.7 years; female age unknown	15-item Chinese (male/female) version	Both male and female participants: Premature voiding (4): 2.41 ± 1.00 Straining to void (4): 1.49 ± 0.88 Place preference for voiding (4): 2.18 ± 1.14 Delayed voiding (3): 2.06 ± 0.74	Never/rarely Sometimes Often/always	Cronbach’s alpha: 0.71 for entire scale	N/A
Xu (2018) [23] China	Participants from an endocrinology department in one hospital who had overactive bladder and Type 2 diabetes mellitus. Men were included in the sample. Overall age: 66.4 ± 8.2 years; female age unknown	15-item Chinese (male/female) version	Both male and female participants: Premature voiding (4): 2.41 ± 1.00 Straining to void (4): 1.49 ± 0.88 Place preference for voiding (4): 2.18 ± 1.14 Delayed voiding (3): 2.06 ± 0.74	Never/rarely Sometimes Often/always	Not reported	N/A
So (2019) [25] Korea	Participants aged 50 years and over who had urinary incontinence Age: 74.35 ± 9.36 years	17-item Korean version	Premature voiding (5) Straining to void (4) Place preference for voiding (3) Delayed voiding (3) Position preference for voiding (2)	N/A	Cronbach’s alpha: 0.78 for entire scale Premature voiding (α = 0.79) Straining to void (α = 0.94) Place preference for voiding (α = 0.81) Delayed voiding (α = 0.83) Position preference for voiding (α = 0.83)	Construct validity: Five subscales explained 74.24% variance of the latent toileting behavior. Concurrent validity: Supported by establishing significant association with responses to the International Consultation on Incontinence Questionnaire Short Form (*r* = 0.146, *p* = 0.011).

Note: N/A indicates not applicable.

## Supplementary Materials

The following are available online at https://www.mdpi.com/1660-4601/16/20/4000/s1, Table S1: Search strategy for each database between January 2010 and July 2019; Table S2: Basic characteristics of the 25 included studies.
