# Supplementary material for: Toileting Behaviors Related to Urination in Women: A Scoping Review
Source: Int J Environ Res Public Health. 2019 Oct 19;16(20):4000. doi: 10.3390/ijerph16204000 (PMC6843934; doi:10.3390/ijerph16204000)
Supplement: Supplementary file 1 [file ijerph-16-04000-s001.zip › supplementary documents/Table S1_10162019FINAL.docx]

**Table S1: Search strategy for each database between January 2010 and July 2019**

| **Database** | **Searching syntax** | **Results** |
| --- | --- | --- |
| **CINAHL** | ( MH "Toileting" OR ti (toilet OR toileting OR toilets OR “voiding behavior*”) OR ab (toilet OR toileting OR toilets OR “voiding behavior*”) OR ((voiding OR micturition OR MH "Urination" OR urinar* OR bladder) AND (habit OR habits)) ) AND ( MH "Women+" OR MH "Female" OR TI (women OR woman OR female OR females) OR AB (women OR woman OR female OR females) ) filter English | 309 |
| **Pubmed** | ((toilet[tiab] OR toileting[tiab] OR toilets[tiab] OR “voiding behavior*”[tiab] OR ((voiding[tiab] OR micturition[tiab] OR urination[mesh] OR urinar*[tiab] OR bladder) AND (habit[tiab] OR habits[tiab]))) AND (Women"[Mesh] OR "Female"[Mesh] OR women[tiab] OR woman[tiab] OR female[tiab] OR females[tiab])) Sort by: Best Match Filters: English | 1692 |
| **Web of Science** | #1 TOPIC: (women OR woman OR female OR females)  #2 TOPIC: (toilet OR toileting OR toilets OR “voiding behavior*” OR ((voiding OR micturition OR urinar* OR bladder) AND (habit OR habits)))  #1 AND #2  Refined by: Language: (English) | 819 |
