# Supplementary material for: Toileting Behaviors Related to Urination in Women: A Scoping Review
Source: Int J Environ Res Public Health. 2019 Oct 19;16(20):4000. doi: 10.3390/ijerph16204000 (PMC6843934; doi:10.3390/ijerph16204000)
Supplement: Supplementary file 1 [file ijerph-16-04000-s001.zip › supplementary documents/Table S2_10162019.docx]

**Table S2. Basic characteristics of the 25 eligible studies**

| First author (year)    Country    Journal | Design | Sample size analyzed (recruited, participated) | Characteristics of female participants  Age^*^ | Toileting behavior measure/tool used | Aim(s) and main finding(s) |
| --- | --- | --- | --- | --- | --- |
| Yang (2010)  Taiwan  *International Urogynecology Journal* | Repeated  measures for uroflowmetry parameters | 45 (51, 51) | University students  Age: 23.2 ± 4.6 years | Field survey  One summarized item to test preferred voiding posture (i.e., sitting, semi-squatting, or crouching) using a public sitting-type toilet and whether particpants felt comfortable when using the selected voiding posture (response:  yes or no) | *Aim 1: To compare the effects of sitting, semi-squatting, and crouching postures using the modified sitting-type toilet^**(1)^ on uroflowmetric parameters and postvoid residual urine volume assessed by ultrasound.*    Women who used the semi-squatting posture^** (2)^ to void had longer “delayed time to void”^**(3)^ than when they used the sitting and crouching postures^**(4)^ (*p* = 0.001 for multiple comparison: semi-squatting > sitting; semi-squatting > crouching); 22.2% had a normal urinary flow pattern (i.e., bell-shaped) using the semi-squatting posture as compared to 51.1% for the sitting posture and 17.8% for the crouching posture. No significant difference in post void residual urine volumes was noted between sitting and the other two postures.    *Aim 2: To investigate women's preferred voiding posture on public sitting-type toilet.*  Semi-squatting and crouching postures were preferred by most women (40/45) when using a public sitting-type toilet. Among women who adopted the semi-squatting posture (*n* = 37), only 32.4% felt comfortable while urinating using a public sitting-type toilet and none who used the crouching posture (*n* = 3) reported feeling comfortable. |
| Wang (2011)  USA  *Nursing Research* | Methodological | 250 (297, 297) | Employees of a large university and academic medical center  Age: 51.2 ± 6.8 years | TB-WEB^***^ (online survey) | *Aim: To develop and validate an instrument called TB-WEB scale.*  An 18-item scale with 5 domains was developed; these five domains are premature voiding (5 items), straining to void (4 items), place preference for voiding (4 items), delayed voiding (3 items), and position preference for voiding (2 items). Cronbach’s alphas for the five domains ranged from 0.70 to 0.88 and all five domains explained 67% variance of the latent toileting behavior. The authors reported that criterion-related validity was confirmed by using a contrasting group approach. |
| Chou (2011)  Taiwan    *Journal of Urology* | Cross-sectional | 21 (26, 26) | Elderly volunteers with knee osteoarthritis  Age: 65.0 ± 4.6 years | Field survey  Questionnaire created by the authors to assess: (1) difficulties women experience when using the standing position to void and 2) willingness to use the standing position after the study and to determine if women would recommend using the standing position to others with similar knee problems. | *Aim 1: To evaluate uroflowmetric characteristics and postvoid residual urine volumes (assessed by ultrasound) among women with knee osteoarthritis when asked to urinate in the standing position using a homemade auxiliary appliance (i.e., paper rolled into a horn shape) to collect urine.*    Maximal and average uroflow rates and voided and post-void residual urine volumes were not statistically different between the sitting and standing positions (*p* > 0.05); uroflow curves were smooth, suggesting that the bladder opening was fully relaxed while in the standing position to void.  *Aim 2: To determine women’s acceptance of the standing position for voiding.*  Most participants (81%) had no difficulty in using the standing position to void and 95% were satisfied with the experience of using the auxillary appliance. All participants expressed their willingness to use the standing position if they did not have access to a clean toilet seat and would recommend this position to others with knee problems. |
| Liu (2012)  China  Neurourology and Urodynamics  (conference abstract) | Methodological | 254 (254, 254) | Living in a community and reported having urinary incontinence  Age: unknown | TB-WEB (field survey) | *Aim: To adapt the English version of the TB-WEB scale for use by Chinese participants.*  A 14-item instrument with four domains was formulated; the domains are: 1) straining to void (4 items), 2) premature voiding (4 items), 3) delayed voiding (3 items), and 4) place preference for voiding (3 items). Cronbach’s alphas for these domains ranged from 0.75 to 0.95. These domains explained 79% variance of the latent toileting behavior. |
| Harvey (2012)    UK  *BJU International* | Qualitative descriptive (focus group with 1 to 5 participants meeting from 1 up to 4 times | 25 (25, 25) | No self-reported urinary problems  Mean age = 60 years, ranging from 21 to 90 years | Semi structured interview  “frequencies/timing, perceived sensation related to the bladder, rationale for going to the toilet, relevance of place and time of day, role of fluid intake, apparent suppression of sensation.” (p. 1757). | *Aim: To investigate the inter-relationship between conscious decision-making processes and bladder sensation in determining the need, time, and place to void.*  Six major themes emerged: (1) temporal maps, (2) cognitive maps, (3) risk issues, (4) habituation, (5) opportunistic behavior, and (6) bladder awareness.  The bladder filling sensation progressively intensified, coupled with a large ‘window’ (i.e., no sensation, awareness, mild sensation, and clear sensation) within which the need to void was not related to the sensation of a full bladder but related to behavioral and social considerations (e.g., voiding immediately before an activity, voiding when an opportunity arose). After the ‘window’, a strong sensation close to the threshold of bladder capacity was linked to the need to void. The authors suggested using the term ‘cognitive voiding’ to describe normal voiding behavior. |
| Palmer (2015)  USA  *International Journal of Clinical Practice* | Cross-sectional survey | 109 (373, 113) | Advanced practice providers (APPs)  Age: 45.1 ± 10.6 years | TB-WEB (online survey) | *Aim: To investigate the toileting behaviors that female APPs use to manage their personal bladder emptying.*  APPs with urinary incontinence were older (47.9 years versus 41.2 years, *t* = -3.3, *p* = 0.001) than APPs who were continent; fewer nulliparous women reported urinary incontinence compared with those who had had one or more pregnancies (34% versus 57%, χ^2^ = 4.5, *p* = 0.03).  Proportionally more APPs with urinary incontinence versus those without urinary incontinence did not avoid using public toilets (27.3% versus 9.6%, *p* = 0.041), fewer APPs with urinary incontinence did not empty their bladders when there was little or no need to urinate (specifically in a public place, 56.4% versus 75%, *p* = 0.043), and proportionally more APPs with urinary incontinence often/always waited too long to urinate (specifically at the workplace, 52.7% versus 26.9%, *p* = 0.006). |
| Xu (2016)    China  *International Journal of Clinical Practice* ^†(1)^ | Cross-sectional survey | 636 (670, 670) | Clinical nurses at three hospitals  Age: 30.6 ± 7.9 years | TB-WEB (field survey) | *Aim 1: To assess the toileting behaviors and health beliefs related to toileting behavior among clinical female nurses.*    Many [65.9% (419)] nurses often/always worried about the cleanliness of public toilets, 53.6% (341) often/always delayed emptying their bladder when busy, 44.2% (281) often/always emptied their bladder with little or no need to urinate before going to sleep, and 32.1% (204) often/always crouched or hovered to empty their bladder when away from home.  *Aim 2: To investigate the relationships between health beliefs and toileting behaviors.*    Perceived barriers (*β* = 0.190, *p* < 0.001), perceived susceptibility (*β* = 0.081, *p* = 0.035), and perceived severity (*β* = 0.146, *p* < 0.001) were significantly associated with toileting behavior. No significant associations were found for perceived benefits (*β* = 0.002, *p* = 0.944) and self-efficacy (*β* = -0.037, *p* = 0.363) associated with toileting behavior. |
| Yang (2017)  Taiwan  *International Journal of Gynecology & Obstetrics* | Cross-sectional | 17 (20, 17) | Pregnant (third trimester)  Age: unknown | Field survey  Questionnaire to evaluate attitudes about voiding in the standing position | *Aim: To evaluate the voiding function of pregnant women when asked to urinate in the standing position with a homemade auxiliary appliance (i.e., paper rolled into a horn shape) to collect urine.*  Post-void residual urine volume assessed by sonography was less in the standing position than in the sitting position (15.8 ml versus 27.5 ml, *p* = 0.003). No significant findings were evident for any uroflowmetric parameters between the two postures; 12 (71%) women were satisfied with using the standing position to void and would use it if a (clean) sitting toilet was unavailable. |
| Wan (2017)  China  *International Journal of Nursing Studies* ^†(1)^ | Cross-sectional survey | 636 (670, 670) | Clinical nurses  Age: 30.6 ± 7.9 years | TB-WEB (field survey) | *Aim 1: To investigate the prevalence of lower urinary tract symptoms among clinical female nurses.*    Many (68%) nurses had at least one lower urinary tract symptom, 50% had incontinence symptoms, 40% had filling symptoms, and 18% had voiding symptoms.  *Aim 2: To investigate the association between toileting behaviors and overall lower urinary tract symptoms.*  Premature voiding (*r* = 0.384, *p* = 0.009), delayed voiding (*r* = 0.501, *p* = 0.007), and straining to void (*r* = 0.672, *p* < 0.001) were significantly associated with lower urinary tract symptoms, i.e., filling and storing, voiding, and incontinence symptoms. |
| Willis-Gray (2017)    USA  *Urologic Nursing* | Cross-sectional survey | 194 (213, 209) | Patients at a urogynecology  clinic  Mean age = 60.4 years | TB-WEB (field survey) | *Aim: To describe toileting behaviors in women who were seeking medical help for urogynecological problems.*  Compared to women without urinary incontinence, those with urinary incontinence were more likely to empty their bladders without having an urge, both at home (47.9% versus 32.1%, *p* = 0.05) and when at someone else’s home (28.8% versus 11.1%, *p* = 0.01). At work, women with urinary incontinence were more likely to wait too long to empty their bladders (41.5% versus 21.6%, *p* = 0.01), strain to start voiding (32.6% versus 16.7%, *p* = 0.03), strain to keep urine flowing (43.9% versus 20.4%, *p* = 0.01), strain to empty their bladder completely (49.3% versus 25.9%, *p* = 0.01), and strain to empty their bladder quickly (37.4% versus 17.0*%, p* = 0.01) compared to those without urinary incontinence.  Women with urinary incontinence were more likely to crouch or hover over the toilet when at home than those who did not report urinary incontinence (17.6% versus 3.8%, *p* = 0.02). |
| Xu (2017)    China    *BMC Urology* | Cross-sectional survey | 1018 (1025, 1025)  Female = 509  Male = 509 | Participants in the endocrinology department in one hospital who had Type 2 diabetes mellitus. Men were included in the sample.  Overall age: 59.1 ± 11.7 years;  female age unknown | TB-WEB (field survey) | *Aim 1: To investigate toileting behaviors that participants used to empty their bladders.*  Sex-specific responses were not reported; 31.1% of participants reported often/always voiding prematurely.  *Aim 2: To investigate the relationships between toileting behaviors and overactive bladder.*  Premature voiding (aOR^‡^ = 1.286, 95% CI: 1.048 ~ 1.579, *p* = 0.016) and straining to void (aOR = 1.243, 95% CI: 1. 026 ~ 1.506, *p* = 0.026) were risk factors for overactive bladder.  Note: aOR is adjusted odds ratio and CI is confidence interval |
| Sjogren (2017)  Sweden    *International Urogynecology Journal* | Cross-sectional survey | 173 (550, 173) | University students  Mean age = 21.6 years, ranging from 18 to 25 years | TB-WEB (postal delivered questionnaire) | *Aim 1: To investigate the association between toileting behaviors and lower urinary tract symptoms.*    At least sometimes, 87.2% of students worried about the cleanliness of public toilets, 45.6% always emptied their bladder at home to avoid using public toilets, 46.5% at least sometimes emptied their bladder without desire at home and ‘just in case’, 69.9% delayed voiding at least sometimes because of work responsibilities, 20.3% strained to void to initiate urinating at least sometimes, 12.7% strained to void during the whole urinating process, 38.8% strained to void at least sometimes to completely empty their bladder, and 42.2% strained to void at least sometimes to empty their bladder quickly. Most students (97.6%) sat down on the toilet at least sometimes. The behavior of hovering over the toilet was reported by 24.4% of women at least sometimes and 4.2% reported squatting on the toilet at least sometimes.  Place preference for voiding, premature voiding, delayed voiding, and straining to void were associated with frequency. Voiding was assessed using the International Consultation on Incontinence Questionnaire-Female Lower Urinary Tract Symptoms (ICIQ-FLUTS) subscales; all toileting behavior subscale scores except for place preference for voiding were significantly related to the ICIQ-FLUTS incontinence subscale.  *Aim 2: To validate the Swedish version of the TB-WEB scale.*  A 19-item scale with five domains was formulated; the domains are (1) premature voiding (5 items), (2) straining to void (4 items), (3) place preference for voiding (4 items), (4) delayed voiding (3 items), and (5) position preference for voiding (3 items). Cronbach’s alphas for the five subscales ranged from 0.71 to 0.84 and explained 66% variance of the latent toileting behavior. |
| Zhou (2018)  USA  *Journal of Women’s Health* ^†(2)^ | Cross-sectional survey | 139 | Employees of a large academic medical center  Age: 46.9 ± 12.9 years | TB-WEB (online survey) | *Aim: To identify factors associated with stages of urinary urgency progression: Stage 1: no urinary symptoms, Stage 2: continent but urinary urgency reported, Stage 3: non-severe urgency urinary incontinence (i.e., incontinent but frequency of leakage ≤* *1/d), and Stage 4: severe urgency urinary incontinence (i.e., frequency of leakage ≥ 1/d).*  Women in Stage 4 had greater odds (aOR^§^ = 6.588, 95% CI: 1.317 – 32.971, *p* = 0.022) of straining to empty their bladder faster than those in Stage 3. |
| Palmer (2018)  USA    *Neurourology and Urodynamics* ^†(2)^ | Cross-sectional survey | 170 (4134, 188) | Employees of a large academic medical center  Age: 47.3 ± 13.6 years | TB-WEB (online survey) | *Aim 1: To describe toileting behaviors that working women often or always use.*  The most commonly reported behaviors that working women often/always used were sitting to urinate at home (98.2%), emptying the bladder completely (88.8%), emptying the bladder before leaving home (80%), and sitting to urinate when away from home (68.8%). About 41% of women often/always delayed voiding when they were busy, and 39.4% often/always worried about the cleanliness of public toilets.  *Aim 2: To investigate toileting behaviors associated with urinary urgency with or without leakage.*  Women who waited too long to urinate while at work had greater odds (aOR^\|\|^ =7.85, 95% CI: 1.57 - 39.24, *p* = 0.012) of having urinary urgency than those who did not wait too long to urinate. |
| Xu (2018)    China  *International Journal of Nursing Studies* | Parallel, pragmatic, open-label randomized trial | 104 (142, 104)  Intervention = 51  Control = 53  Female = 58  Male = 46 | Participants in an endocrinology department in one hospital who had overactive bladder and Type 2 diabetes mellitus. Men were included in the sample.  Overall age: 66.4 ± 8.2 years; female age unknown | TB-WEB (field survey) | *Aim: To investigate whether an education program that targets toileting behaviors and is guided by the Health Belief Model is effective for helping patients with Type 2 diabetes and overactive bladder adopt healthy toileting behaviors, improve bladder symptoms, and enhance quality of life.*  Compared with the control group, significant average marginal effects (joint effects of the intervention and interaction between intervention and time) were observed among premature voiding (-0.7, *p* < 0.001), place preference for voiding (-0.5, *p* = 0.007), and delayed voiding (-0.2, *p* = 0.011). The average marginal effects demonstrated that the intervention significantly relieved bladder symptoms (-2.2, *p* < 0.001) and minimized the probability of developing wet overactive bladder, i.e., urgency urinary incontinence (-0.3, *p* < 0.001), decreased the severity of urgency (-0.4, *p* < 0.001), and improved the overactive bladder-specific quality of life (10.8, *p* = 0.001) and general quality of life (3, *p* = 0.049). |
| Shannon (2018)    USA    *Urologic Nursing* | Cross-sectional survey | 200 (400, 200) | Clinical nurses  Age: 40.3 ± 12.5 years | Two-page field survey in three parts: (1) descriptive items, including number of breaks per day and voids per shift, (2) two multiple choice items about toileting habits and bathroom access at work, and (3) UDI-6^¶^ | *Aim: To describe the toileting behaviors of nurses across several nursing units at an urban tertiary care medical center.*  Most nurses (88%) reported that they needed to delay voiding, 15.5% said they used the toilet as soon as they got the urge, and 29.5% reported that there were not enough bathrooms on their unit. |
| Kowalik (2019)    USA  *Journal of Urology* | Cross-sectional survey | 6695 (106,074, 6695) | Living in a community  Age: 41.4 ± 15) years | TB-WEB (online survey) | *Aim: To assess toileting behaviors in community-dwelling adult women.*  Women who reported perceived bladder problems were more likely to report behaviors of convenience (i.e., premature) voiding, delayed voiding, and straining to void; 98.8% (*n* = 6613) of women demonstrated at least one place preference behavior for voiding; 85.1% (*n* = 5700) women delayed emptying their bladders when busy; and 53.1% (*n* = 3552) waited to void until they could not hold urine any longer. At home, 99.4% (*n* = 6657) of women sat to void. When they were away from home, this percentage decreased to 76.3% for women who reported sitting at least sometimes and 11.9% (*n* = 796) for women who never sat on public toilets. |
| So (2019)    Korea  *International Neurourology Journal* | Methodological | 321 (330, 330) | Aged 50 years and over who had urinary incontinence  Age: 74.35 ± 9.36 years | TB-WEB (field survey) | *Aim: To adapt the English version of TB-WEB scale for use in Korean participants and test its reliability and validity among older Korean women with urinary incontinence.*  A 17-item instrument with five subscales was formulated; the subscales are: (1) place preference for voiding (3 items), (2) premature voiding (5 items), (3) delayed voiding (3 items), (4) straining to void (4 items), and (5) position preference for voiding (2 items). Cronbach’s alpha of these subscales ranged from 0.75 to 0.95, and the overall Cronbach’s alpha was 0.78. These subscales explained 74.24% variance of the latent toileting behavior. Concurrent validity was established by significant association with the International Consultation on Incontinence Questionnaire Short Form (*r* = 0.146, *p* = 0.011*).* |
| Pierce (2019a)  Australia  *Journal of Advanced Nursing* | Qualitative descriptive (focus group) | 96 (96, 96) | Clinical nurses and midwives  Age: 42.3 ± 12.6 years | Semi-structured questions | *Aim: To provide an in-depth exploration of participants’ experiences of urinary symptoms while at work.*  Participants’ experiences of urinary symptoms while at work were related primarily to delayed voiding. This behavior was explained by multiple work-related factors, including the ‘patient first’ work culture, relationships in the nursing team, demands of the nursing role, and inadequacy of workplace amenities. |
| Xu (2019)    China  *Journal of Advanced Nursing* | Cross-sectional survey | 381 (450, 400)  Female = 320  Male = 61 | Operating room nurses with males included in the sample.  Overall age: 30.2 ± 7.1) years;  female age unknown | TB-WEB (field survey) | *Aim 1: To investigate the prevalence of overactive bladder (diagnosed using overactive bladder symptom score questionnaire, with urgency symptom score ≥ 2 and a total score ≥ 3) among male and female operating room nurses*.  One in three nurses experienced overactive bladder.    *Aim 2: To investigate the toileting behaviors that nurses used to void their bladders*.    Sixty-one percent of nurses often/always delayed voiding; 52% of nurses often/always worried about the cleanliness of public toilets, and 33% often/always avoided using public toilets. Thirty-nine percent of the nurses often/always emptied their bladders with no need to urinate before going to sleep (39%), and 22% often/always emptied their bladders with no need to urinate before leaving home.  *Aim 3: To investigate the mediating roles that different toileting behaviors play in the relationship between occupational stress and overactive bladder.*  Toileting behaviors fully mediated the relationship between occupational stress and overactive bladder. With high levels of occupational stress, nurses tended to delay voiding (*β_1a_* = 0.015, *p* < 0.001) and strained to void (*β_2_* = 0.009, *p* < 0.05) frequently. The more frequently that nurses delayed voiding (*β_1b_* = 0.383, *p* < 0.05) and strained to void (*β_2b_* = 0.351, *p* < 0.05), the greater the likelihood of having an overactive bladder. |
| Angelini (2019)  USA  *Female Pelvic Medicine & Reconstructive Surgery* | Cross-sectional survey | 166 (500, 193) | Private college undergraduate students  Age: 21.2 ± 0.46 years | TB-WEB (online survey) | *Aim 1: To investigate toileting behaviors among participants.*  The frequency of toileting behaviors was categorized according to rating: rarely, sometimes, often, and always. Most participants worried about the cleanliness of public toilets (92.2%; *n* = 153), 83% (*n =* 138) reported that they tried to avoid public toilets, 97% (*n =* 161) emptied their bladders before leaving home, 81.9% (*n =* 136) reported trying to hold their urine until they got home, 95.2% (*n* = 158) reported that they delayed emptying their bladders when they were busy, and 72.3% (*n =*120) reported straining in order to empty their bladder quickly.  *Aim 2: To assess the psychometric properties of the TB-WEB scale when used with a sample of college women.*  An existing 18-item scale with five domains was used. The domains are (1) premature voiding (5 items), (2) straining to void (4 items), (3) place preference for voiding (4 items), (4) delayed voiding (3 items), and (5) position preference for voiding (2 items). The Cronbach’s alpha for position preference for voiding was 0.582 and ranged from 0.731 to 0.919 for the other domains; all domains explained 72.8% variance of the latent toileting behavior. Construct validity was confirmed by establishing significant associations between toileting behaviors and stress urinary incontinence (*r* = 0.293, *p* = 0.015) and urgency urinary incontinence (*r* = 0.342, *p* < 0.001). Stress urinary incontinence was related to sport or physical activity (*r* = 0.350, *p* < 0.001) and urinary urgency (*r* = 0.334, *p* < 0.001). |
| Palmer (2019)  USA  *BMC Women’s Health* | Qualitative descriptive (focus group) | 24 (24, 24) | With or without urinary incontinence or overactive bladder  Age: 68 ± 13.4 years | A discussion guide that reflects constructs of TB-WEB scale, including seven main questions with associated probes | *Aim 1: To describe women’s understanding of bladder health.*  Most women expressed difficulty in describing bladder health. Several informative responders talked about bladder function, presence or absence of bladder symptoms, and control over the bladder as proxies for bladder health.    *Aim 2: To understand why women used various toileting behaviors at home or away from home.*  Internal or external cues/triggers/alerts, toilet cleanliness, regarding toileting as an irritant and a nuisance, and situational awareness helped explain reasons for women’s various toileting behaviors. |
| Pierce (2019a)  Australia  *Journal of Advanced Nursing* | Qualitative descriptive (focus group) | 96 (96, 96) | Clinical nurses and midwives  Age: 42.3 ± 12.6 years | Semi-structured questions | *Aim: To provide an in-depth exploration of participants’ experiences of urinary symptoms while at work.*  Participants’ experiences of urinary symptoms while at work were related primarily to delayed voiding. This behavior was explained by multiple work-related factors, including the ‘patient first’ work culture, relationships in the nursing team, demands of the nursing role, and inadequacy of workplace amenities. |
| Pierce (2019b)  Australia    *Journal of Advanced Nursing* | Cross-sectional study | 353  (600 handouts plus unknown number via online access, 365) | Female nurses and midwives  Age: 42.3 ± 12.6 years | Field survey and online survey  Questions devised by authors about:  1) Whether participants could access the toilet whenever required (never, occasionally, and sometimes indicate limited access).  2) How often participants delayed passing urine at work.  3) How often participants reduced fluid intake to delay or avoid passing urine at work (sometimes, most of the time, or all of the time indicate delayed voiding or limited fluid intake). | *Aim 1: To describe the proportion of participants who have restricted access to toilets, delayed voiding, or purposefully limited their fluid intake to delay voiding.*  At work, 22.4% of participants could not access a toilet when needed, 77.1% reported delaying voiding, and 26.9% reported limiting their fluid intake to delay voiding at work. Participants who delayed voiding were more likely to report urinary symptoms than those who did not delay voiding.    *Aim 2: To describe urinary symptoms, including bladder discomfort, and/or storage lower urinary tract symptoms among participants.*    The prevalence of overall urinary symptoms was 46.7 percent.    *Aim 3: To explore the relationship between restricted access to toilets, delayed voiding, and limited fluid intake and the ability to do their job among participants with urinary symptoms at work.*  Participants who reported restricted access to toilets or reported limiting their fluid intake while at work were more likely to have physical work impairments (6.12 versus 1.71, *p* = 0.004; 9.69 versus 3.76, *p* = 0.013). Participants who delayed voiding at work were more likely to show concentration impairment (20.73 versus 11.87, *p* = 0.018) and those who limited their fluid intake at work were more likely to show time management impairment (11.06 versus 6.24, *p* = 0.054). |
| Reynolds (2019)  USA  *The Journal of Urology* | Cross-sectional | 3062 (106000, 7892) | Full-time working American women  Age: 38.7 ± 12.2 years | TB-WEB (online survey) | *Aim1: To understand reasons why participants purposefully limited restroom use at work.*  1,668 (54%) of participants reported that they purposefully limited restroom use at least occasionally. Lower satisfaction with restrooms in workplaces, especially with regard to restroom cleanliness and lack of privacy (no statistical inference), poor quality of restrooms (40% versus 23%, *p* < 0.001), limited availability of restrooms (14% versus 10%, *p* = 0.05), and employer restrictions (7% versus 2%, *p* < 0.001) were reported by proportionally more participants who limited their restroom use than those who did not limit restroom use.    *Aim 2: To describe the bladder habits of participants who purposefully limited restroom use at work.*    Of the participants who purposefully limited restroom use, 84.4% worried about the cleanliness of public toilets, 72.5% avoided using public toilets, and 63.8% held urine until they got home. Moreover, 84.6% of the participants who limited their restroom use delayed urination when busy, 49.3% waited until they could not hold urine any longer, and 47.8% hovered over the toilet to urinate when away from home. All these percentages were higher for participants who reported limited restroom use than for those who did not limit their restroom use.    *Aim 3: To explore whether participants who limited their restroom use at work reported more lower urinary tract symptoms and fewer urinary tract conditions (urinary urgency, urge urinary incontinence, overactive bladder, stress urinary incontinence, monthly urinary incontinence, recurrent urinary tract infection ≥ 3 times in the past year, and voiding < 7 times per day) compared with those who did not limit their restroom use at work.*  Participants who limited their restroom use at work had a higher total urinary symptom score, voiding subscale score, and urinary incontinence score after controlling for all covariates. Urinary urgency, stress urinary incontinence, and monthly urinary incontinence, and voiding fewer than 7 times per day were significantly associated with purposefully limited restroom use after controlling for all covariates. |
| Zhou (2019)  China    *International Urogynecology Journal* | Cross-sectional survey | 929 (1000, 1000) | College and university students  Age: 20.5 ± 1.6 years | TB-WEB (field survey) | *Aim 1: To identify the prevalence of urinary incontiennce among college and university students.*  The overall prevalence of urinary incontinence was 23.6%.  *Aim 2: To explore the association between toileting behaviors along with other factors and urinary incontinence among this group of population.*  Many *(*72.2%) women reported that they habitually (rated as ‘often’ or ‘always’) emptied their bladder before leaving home, 52.7% habitually worried about the cleanliness of public toilets, 36.4% habitually tried to avoid using public toilets, and 25.3% habitually delayed emptying their bladder when busy. Less than 20% of women reported that they often or always used other types of toileting behaviors. Those who habitually delayed voiding (OR = 1.738, 95% CI: 1.306 - 2.313, *p* < 0.001) and strained to void (OR = 1.433, 95% CI = 1.111-1.849, *p* = 0.006) increased the odds of reporting urinary incontinence. Women aged 21 to 26 years had lower odds of urinary incontinence compared to women aged between 18 to 20 years old (OR = 0.867, 95% CI: 0.711 - 0.975, *p* = 0.017). Constipation (OR = 2.395, 95% CI = 1.494 - 3.839, *p* < 0.001) and alcohol consumption (OR = 1.763, 95% CI = 1.114 - 2.792, *p* = 0.016) increased the odds of having urinary incontinence. |

Notes:

^*^Age = (mean age ± standard deviation), unless otherwise noted; ^**(1)^“The sitting-type toilet was modified by adding a wooden frame with a 7.5-cm flatform on both sides;” ^**(2)^“Semi-squatting posture refers to the posture with individuals’ feet on the floor and their buttocks not making contact with the toilet seat;” ^**(3)^“Delayed time to void refers to the elapsed time from ‘begin to urinate’ to ‘actually urinate;’” and ^**(4)^ “Crouching posture refers to the posture with individual’s feet on the toilet seat and squatting over the toilet bowl.” ^***^TB-WEB is the Toileting Behaviors: Women’s Elimination Behaviors scale.^†(1)^^, †(2)^ Two studies used the same dataset; ^‡^ adjusted for demographic and health-related variables; ^§^ adjusted for losing urine with defecation; ^||^ adjusted for age and use of panty liners for urinary leakage; ^¶^ UDI-6 is the Urogenital Distress Inventory Short Form.
